# The Maryland Dental Law

**Published:** 1886-07

**Authors:** Richard Grady

**Affiliations:** Secretary of State Board of Dental Examiners.


ARTICLE VIII.

### THE MARYLAND DENTAL LAW.

BY RICHARD GRADY, D. D. S., SECRETARY OF STATE BOARD OF DENTAL EXAMINERS.

The amendments to the Maryland Dental Law have just been published. The original law passed by the Maryland Legislature in 1884 called forth criticisms from the Pennsylvania State Dental Examining Board, the National Association of Dental Examiners and several dental journals. Two bills were before the Legislature of 1884, one emanating from the Maryland State Dental Association, and the other presented by the Dental Legislative Society. Neither bill was adopted in its entirety, but it is only just to record the fact that neither bill contained the restrictive provision, noted in the protest of the Pennsylvania Dental Examiners, *that it shall be unlawful for any one to engage in the practice of dentistry unless he shall first have passed a satisfactory examination before the Board of Examiners or shall hold a diploma from a university or college chartered by or under the laws of Maryland.*

When the Pennsylvania Dental Examiners filed their protest, the original paper was forwarded by the Maryland

Board of Dental Examiners to the Attorney-General of the State who was asked to construe the law. The Attorney-General never responded to this request, but the Governor of the State, himself a lawyer, in a personal interview, suggested that the Board continue to issue temporary certificates to graduates of reputable dental colleges outside of the State, which could be renewed every six months, and promised to second an effort to have the law amended by the Legislature of 1886.

In the meantime the National Association of Dental Examining Boards adopted the following:

"Whereas the dental law of the State of Maryland seems to be restrictive in its character; it is the sense of this body that the dental profession of said State of Maryland, at the next session of its Legislature, should seek to cause said dental law to be amended so as to be in harmony with the dental laws of the other States."

Acting upon this, the Dental Examiners of Maryland called a mass meeting of the dental profession of the State and submitted the following amendments, which after discussion, were unanimously adopted as the sense of the meeting:

*Be it enacted by the General Assembly of Maryland*, That sections one and eight of the act passed at the January session, eighteen hundred and eighty-four, entitled "AN ACT TO INSURE THE BETTER EDUCATION OF PRACTITIONERS OF DENTAL SURGERY, AND TO REGULATE THE PRACTICE OF DENTISTRY IN THE STATE OF MARYLAND" be and the same are hereby repealed and re-enacted, so as to read as follows.

SECTION 1. That it shall be unlawful for any person who is not at the time of the passage of this act, engaged in the practice of dentistry, to practice dentistry, unless he or she shall have obtained a certificate as herein provided, or shall hold a diploma from a university or college authorized to grant diplomas in Dental Surgery; any person holding such a diploma, and desiring to commence such practice, shall present the same to the Board of Examiners created by this act, for approval; such Examining Board, being satisfied as to the qualifications of the applicant and the genuineness of the diploma, shall endorse the same as approved, and issue the certificate of registration provided for in this act.

SECTION 8. That nothing shall be so construed as to interfere with the rights and privileges of resident physicians and surgeons in the discharge of their professional duties.

The Legislature of 1886 on the very last day of its session repealed sections one and eight of the law of 1884 and re-enacted them as recommended by the profession without the change of a single word.

It may be remarked by way of parenthesis, that neither the Dean of the Dental Department, of the University of Maryland, nor the Dean of the Baltimore College of Dental Surgery, regarded as generous or just the law that denied recognition to diplomas granted in other States, for the one, although not present at the mass meeting, wrote in regard to the amendments: "I *heartily approve* of them and desire to see the bill passed by the Legislature;" and the other who participated in the meeting spoke in favor of them and contributed to make the vote unanimous.

The terms of office of two members of the Board, Drs. Waters and Nelson, having expired, they were re-appointed by Gov. Lloyd, so that the members of the Board continue as originally appointed by Gov. McLane, now Minister to France; namely, Dr. E. P. Keech, president, Dr. Richard Grady, secretary, Dr. T. S. Waters, Dr. Charles E. Duck, all of Baltimore, and Dr. Edward Nelson, of Frederick.

